# Factors for choosing a pediatric dentist in Saudi Arabia

**DOI:** 10.6026/973206300191411

**Published:** 2023-12-31

**Authors:** Ateet Kakti, Rahaf Ali Bin Salamah, Farah Zaid Alhamdan, Bashar Ayed Alanazi, Balsam Dawood Alghomlas, Azzam Abdulrahman Al Saleh, Abdulmajeed Abdullah Alhasmi

**Affiliations:** 1 Riyadh Elm University, Riyadh, Kingdom of Saudi Arabia; 2 General Dentist, Riyadh, Saudi Arabia; 3 King Saud Bin Abdulaziz University for Health Sciences, Riyadh, Kingdom of Saudi Arabia; 4 Prince Sattam Bin Abdulaziz University, Al-Kharj, Kingdom of Saudi Arabia; 5 King Abdulaziz Medical City National Guard Hospital, Riyadh, Saudi Arabia; 6 MOH, Riyadh, Saudi Arabia; 7 Periodontic Division, King Salman Hospital, MOH, Riyadh, Saudi Arabia

**Keywords:** Pediatric dentist, parental decision, dental treatment concerns, dental visit history, Saudi Arabia

## Abstract

The choice of a pediatric dentist is a crucial decision for parents, influenced by a variety of factors. This study aimed to investigate the key determinants that guide parents in the Kingdom of Saudi Arabia in their selection of a pediatric dentist. A cross-sectional survey was administered to a diverse participant pool. The survey captured demographic information, history of dental visits, parents' level of concern about dental treatment for their children, and the importance of specific factors in the choice of a pediatric dentist. The survey was completed by a balanced representation of different age groups and genders. Most participants from Riyadh and other regions had a history of dental visits. Parents' concern about dental treatment varied based on the number of children they had. The most significant factors influencing the choice of a pediatric dentist were the quality of the dental unit's disinfection process, the dentist's ability to communicate with the child and manage uncooperative behaviour, and the dentist's experience. The least influential factors were the proximity of the dental office to the child's school, the dentist's gender, and the number of pediatric dentists in the practice. This study provided valuable insights into the factors that influence parents' decision in choosing a pediatric dentist in The Kingdom of Saudi Arabia. Data helps dental professionals understand parents' priorities and improve their services accordingly to meet patient needs and expectations.

## Background:

Pediatric dentistry plays a crucial role in maintaining children's oral health and preventing dental diseases. Parents play a significant role in choosing a pediatric dentist for their children, it can be a daunting task and various factors may influence their decision. In the Kingdom of Saudi Arabia (KSA), where the prevalence of dental caries among children is high as evidenced by a recent study published in 2022, it revealed that the prevalence of caries among school children in Saudi Arabia was 84% for 5-7 years' children, and 72% for 12-15 years' children [1]. Several other studies [2, 3,4] have identified factors that parents consider when choosing a pediatric dentist, such as the quality of care, the dentist's competence, their ability to explain the treatment and involve parents in the decision-making process, additional qualifications of the pediatric dentist and recommendations from relatives, friends and acquaintances for the choice of a pediatric dentist. Additionally, factors like the appearance and gender of the dentist may also come into play [4]. This branch of dentistry is not merely a matter of applying the principles of adult dentistry to a child [5]. Children have unique dental needs and issues, such as dealing with the changes associated with primary (baby) teeth and then the transition to permanent teeth [6]. Additionally, children may have behavioral or emotional needs that require a specific approach to ensure positive experiences and relationships with dental health from an early age. Moreover, the introduction of good oral hygiene habits from an early age is a critical aspect of pediatric dentistry [7,8,9]. A pediatric dentist does not only treat oral health issues, but they also educate children and parents on how to prevent dental issues through proper oral hygiene and dietary habits [2,10]. This educational role can significantly impact a child's long-term oral health and can influence parents' decision in choosing a pediatric dentist. Additionally, pediatric dentistry plays a crucial role in diagnosing and treating oral conditions related to other health issues like diabetes, congenital heart defects, asthma, and attention deficit/hyperactivity disorder (ADHD) [11]. A pediatric dentist with knowledge and experience in dealing with these specific health concerns can be a valuable asset for families dealing with these conditions [12-13]. However, these factors may vary depending on the cultural, social, and economic factors in different countries. Therefore, it is of interest to investigate the factors that influence parents' decision-making when choosing a pediatric dentist specifically in KSA. By identifying the factors that influence parents' decision-making when choosing a pediatric dentist in KSA, dental professionals can improve their services and provide better care for children. Moreover, data helps policymakers develop appropriate strategies to promote children's oral health in KSA.

## Materials and Method:

## Study design:

This study employed a cross-sectional design, a type of observational study that analyzes data from a population, or a representative subset, at a specific point in time. It was conducted online, which allowed for a broader geographic reach and convenience for the respondents. The goal was to explore the factors that influence the selection of pediatric dentists by residents in Saudi Arabia.

## Participants:

The study involved 532 participants, comprising 221 males and 311 females. This gender distribution might reflect the general demographic breakdown in the population or the differential response rates by gender. The participants were residents of Saudi Arabia, a criterion that ensured the study's findings were representative and applicable to the Saudi context. The questionnaire was distributed via social media, a strategy that enabled easy and quick access to a large audience.

## Questionnaire design:

The questionnaire was adapted from a previous study by Mourad *et al.* [3], with minor modifications to suit the context of this particular study. Incorporating a pre-existing questionnaire helped maintain some level of scientific rigor and comparability since the original instrument had presumably undergone validation processes.

## Section A:

## Demographic information:

The first portion of the questionnaire sought to collect demographic information. This data is crucial in understanding the background of the respondents, which can potentially influence their perception and choice of pediatric dentists. Questions about whether the respondent had previously taken their children to a dentist, and their concerns about dentists' treatment of preadolescent patients, offer insights into the past experiences that might influence their choices and preferences.

## Section B:

## Influencing factors:

The second section aimed to identify specific factors that influence the choice of a pediatric dentist. The use of a 5-point scale offers a degree of granularity, allowing for a nuanced understanding of the factors' relative importance.

## Data management and analysis:

The collected data was managed and analyzed using the Statistical Package for the Social Sciences (SPSS) software (version 21, Chicago, Illinois, USA). SPSS is a widely used tool for statistical analysis in social science research, offering various functions for managing and analyzing data. We employed a stratified random sampling method, which helps ensure that the sample is representative of the population, thereby increasing the accuracy and reliability of the study. In this study, a p-value of less than or equal to 0.05 was considered statistically significant, a commonly accepted threshold in many scientific studies. This means that if the study's results have a p-value of less than or equal to 0.05, the results are unlikely to have occurred by chance, supporting the study's hypotheses.

## Results:

A total of 532 responses were collected from the questionnaire that included all regions of Saudi Arabia. This is the first study in Saudi Arabia that investigates the most significant factors that affect the parents' decision when it comes to choosing a pediatric dentist. The administered questionnaire was divided into three distinct sections. The initial section focused on the demographic data of the participants and their geographical distribution, with a particular emphasis on the central region. The remaining sections emphasized the importance of dentist qualifications and privileges, logistical considerations in selecting pediatric dentists, and the impact of word-of-mouth recommendations.

**Figure 1:**
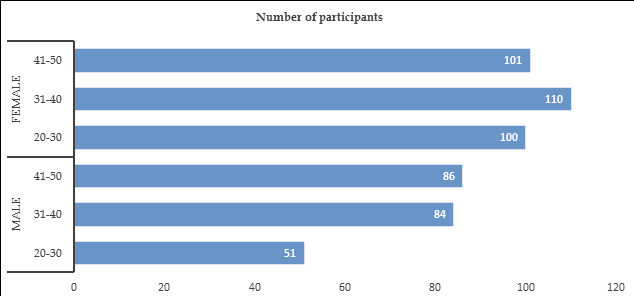
Graphical representation of the gender and age distribution of the assessed sample size

The data in Table 1 and Figure 1 respectively detail the gender and age distribution of the participants in the study. Among the male participants, there were 50 individuals (9.4% of the total) in the 20-30 years age group, 85 individuals (16.0% of the total) in the 31-40 years age group, and 86 individuals (16.2% of the total) the 41-50 years age group. In terms of female participants, there were 100 individuals (18.8% of the total) in the 20-30 years age group, 110 individuals (20.7% of the total) in the 31-40 years age group, and 101 individuals (19.0% of the total) in the 41-50 years age group. The Mean, SD, and p-value columns were not filled in this table, as these statistical measures did not apply to the categorical variables of gender and age group.

**Figure 2:**
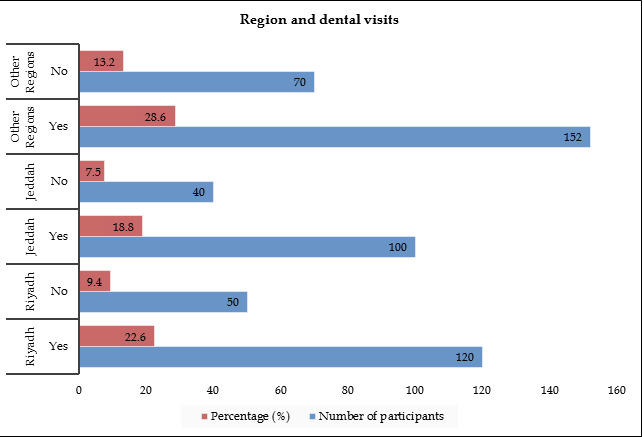
Graphical representation of the participants' residency and previous dental visits

Table 2 and Figure 2 respectively provide information about the participants' residency and their history of dental visits. Among those who resided in Riyadh, 120 participants (22.6% of the total) had visited a dentist previously, while 50 participants (9.4% of the total) had not. For those living in Jeddah, 100 participants (18.8% of the total) had a previous dental visit, and 40 participants (7.5% of the total) did not. Among the participants from other regions, 152 individuals (28.6% of the total) had visited a dentist before, whereas 70 individuals (13.2% of the total) had not. Similar to Table 1, the Mean, SD, and p-value columns were left blank in this table as these measures were not applicable to the categorical variables of residency region and previous dental visits.

The data (Table 3) reflected details about the number of children each participant had and their associated level of concern about dentists' treatment. For families with only one child, it was observed that 20 participants (3.8% of the total) had no concern (level 1) about dental treatment for their child. The mean concern level for this group was recorded as 1.0 with no standard deviation, indicating no variation in their responses. Additionally, 30 participants (5.6% of the total) who had one child expressed a moderate level of concern (level 3) about dental treatment. Their responses also showed no variation with a mean level of 3.0 and a standard deviation of 0.0.In the same one-child family group, 40 participants (7.5% of the total) had a high level of concern (level 5) about dental treatment. Again, there was no variation in their responses, with a mean concern level of 5.0 and a standard deviation of 0.0.

Among participants with 2-3 children, 32 (6% of the total) had no concern (level 1), while 88 (16.5% of the total) had a moderate level of concern (level 3) and 60 participants (11.3% of the total) expressed a high level of concern (level 5). In all these categories, the responses were consistent, with a mean that matched the concern level and a zero-standard deviation. Interestingly, none of the participants with more than four children expressed either no concern (level 1) or a high level of concern (level 5). However, there were 50 participants (9.4% of the total) in this group who had a moderate level of concern (level 3). Their mean concern level was 3.0, with no standard deviation, indicating a unanimous level of concern among these participants.

**Figure 3:**
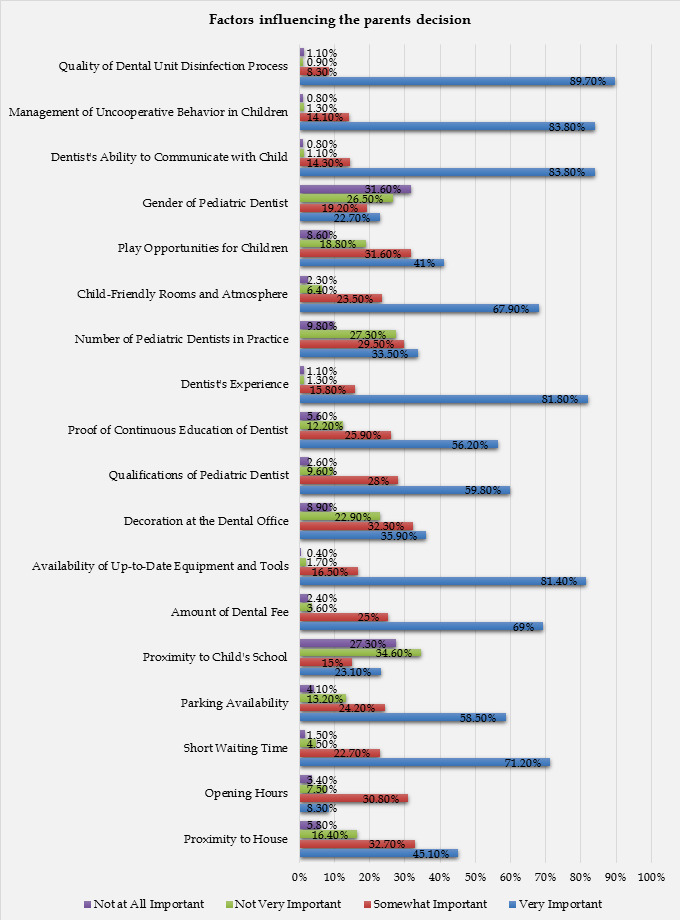
Graphical representation of the factors influencing the parents' decision

As elucidated through Table 4 and Figure 3 respectively, several factors were examined regarding their importance in the selection of a pediatric dentist. The proximity of the dental office to the house was considered very important by 45.1% of respondents, somewhat important by 32.7%, not very important by 16.4%, and not at all important by 5.8%. The opening hours of the dental clinic were seen as less significant; with only 8.3% finding them very important. Short waiting times were a key issue for many respondents, with 71.2% considering them very important. Also, the availability of parking was very important to 58.5% of those surveyed. The proximity of the dental office to the child's school was less influential, with only 23.1% finding it very important. The amount of the dental fee was seen as very important by 69% of participants, and the availability of up-to-date equipment and tools was considered very important by a significant 81.4% of respondents. The decoration at the dental office held less sway, with 35.9% rating it as very important.

The qualifications of the pediatric dentist were seen as very important by 59.8% of respondents, and proof of the dentist's continuous education was very important to 56.2% of respondents. The dentist's experience was a crucial factor for a large majority of respondents, with 81.8% marking it as very important. The number of pediatric dentists in the practice was less influential, with only 33.5% considering it very important. A child-friendly atmosphere in the clinic was deemed very important by 67.9% of respondents. Meanwhile, play opportunities for children were considered very important by 41% of participants. The gender of the pediatric dentist was the least influential factor, with only 22.7% rating it as very important. The dentist's ability to communicate with the child and manage uncooperative behaviour was both considered very important by 83.8% of respondents. The most significant factor was the quality of the dental unit's disinfection process, which was found to be very important by 89.7% of participants.

## Discussion:

Our findings regarding dentist qualifications and privileges revealed that the most highly valued criterion by the participants was the quality of the dental unit disinfection process. This was closely followed by the dentist's ability to communicate effectively with the child and the dentist's overall experience. The least important criteria, according to participants, were the gender of the pediatric dentist, the number of pediatric dentists in the practice, and evidence of the dentist's ongoing education. With respect to logistical considerations, our descriptive analysis revealed that the availability of up-to-date equipment and tools at the clinic was the most significant factor, with the majority of respondents marking it as 'very important'. This was followed by the importance of short waiting times, and then the amount of dental fee. The proximity of the dental clinic to the child's school, the decoration of the dental office, and the clinic's proximity to the residence were deemed the least important criteria. Moreover, in terms of word-of-mouth recommendations, parents found educational institutions such as schools and internet portals to be the least trustworthy sources. Formal criteria, such as the dentist's gender, were considered 'not important' and were perceived as mere information rather than a determining factor in the decision-making process.

In a series of recent investigations conducted as per the same objectives as ours, researchers executed an array of surveys with the intent to comprehend the significant attributes taken into account during the selection process of a pediatric dentist. The outcomes of our investigation were juxtaposed with those of two analogous studies previously conducted within the region of Saudi Arabia conducted in 2020 [5] and 2012 [12] respectively. Our analysis revealed that the most salient factor patients take into consideration when selecting a pediatric dentist is the dentist's professional experience. 81.8% of respondents attributed high importance to this characteristic. This finding is consistent with the 2012 study, which reported a statistic of 87.8% and the 2020 study, which reported a slightly lower percentage of 78%. Another pivotal factor that patients contemplate is the quality and effectiveness of the dental unit's disinfection process. According to our investigation, a significant 89.7% of the respondents deemed this as an important factor. In the 2012 study [12], a comparable percentage of 84.2% of respondents also indicated this to be a crucial consideration. However, the 2020 study [5] did not provide any data regarding this particular factor.

Data also shows that the additional qualifications held by the pediatric dentist are a consideration during the selection process, with 59.8% of respondents indicating this as an important factor. This statistic, however, is marginally lower than the 65.9% reported in the 2020 study, which concentrated specifically on the factors influencing parents' decision when choosing a pediatric dentist. Comparing the findings with those of Kopczynski *et al.* [11] reveals some notable distinctions and parallels. One of the significant differences is the focus of the two studies. Factors influencing the selection of a pediatric dentist, with less emphasis on the specific treatment decisions once a dentist was chosen. On the other hand, Kopczynski *et al.* [11] focused on the treatment decisions, specifically the choice between Silver Diamine Fluoride (SDF) treatment, conventional restorative treatment under local anesthesia, and restorative treatment under general anesthesia. Despite these divergent focuses, both studies underscore the importance of child-related factors in decision-making processes. Significant emphasis was placed on the dentist's ability to manage uncooperative behaviour, suggesting that the child's comfort and behaviour were critical considerations for parents. Similarly, Kopczynski *et al.* [11] found that child dental anxiety was the primary factor associated with treatment decisions, reinforcing the notion that child-centric factors are integral to these decisions. Moreover, both studies identified differences across various demographic factors. We found variations based on the number of children in the family and the region of residency. They also noted significant differences across treatment groups in terms of child age, parent education level, family income, dental insurance status, dental visit behavior rating, and DMFT. However, when included in a multivariate analysis, only child dental anxiety remained a significant covariate, highlighting the complexity of these decision-making processes.

Most participants had a history of dental visits, but the data did not specify when these visits began. Similarly, Kochar *et al.* [13] found that only 8% of parents took their child to the dentist when their first tooth erupted, suggesting that most parents were not aware of the need for early dental visits. Both studies also highlight the importance of the dentist's communication skills. Dentist's ability to communicate with the child was a key factor in parents' choice of a pediatric dentist. Kochar *et al.* [13] reported that 56% of children had bad dental experiences, suggesting a need for better dental care and improved communication. Factors such as the proximity of the dental office to the child's school and the dentist's gender were less influential in the choice of a pediatric dentist. Meanwhile, Kochar *et al.* [13] found that 31% of parents considered the dental clinic's location as an important factor. This discrepancy may be due to cultural differences or unique regional circumstances. They also reported specific behaviours such as prolonged pacifier use and avoidance of regular dental check-ups that were not covered. These factors, particularly the avoidance of regular dental visits, could be significant in shaping parents' attitudes and behaviours towards pediatric dental care. It should be noted that we did not explore the children's reactions to dental visits as Kochar *et al.* [13] did. Their finding that 40% of children were scared and reluctant during their first dental visit could be an essential factor to consider in future studies, particularly in understanding how to improve the child's dental experience and manage uncooperative behaviour.

Despite the valuable insights generated by this study, certain limitations must be acknowledged. Firstly, the study's cross-sectional design only provides a snapshot of the factors influencing parental decisions at a particular point in time. As attitudes and opinions can evolve, a longitudinal approach might have provided a more comprehensive understanding of changes in these determinants over time. Secondly, the reliance on self-reported data introduces the potential for response bias. Participants may have been inclined to provide socially desirable responses, especially when discussing sensitive topics such as concern about their children's dental treatment, potentially skewing the results. Thirdly, while the study achieved a balanced representation of different age groups and genders, the regional distribution appears skewed towards Riyadh and other regions. This geographic bias could limit the generalizability of the findings to the entire Kingdom of Saudi Arabia. Furthermore, the study used a predefined set of factors for selecting a pediatric dentist, which may not have encompassed all possible considerations for every participant. The use of open-ended questions or qualitative interviews could have allowed for the emergence of additional, unanticipated factors.

## Conclusion:

Data shows that the region of residency and the history of dental visits played an essential role in shaping participants' experiences and attitudes towards dental care. A spectrum of responses was observed, with variations based on the number of children in the family. This underlines the importance of individualized communication strategies to address the unique concerns of different family types. The study, more importantly, shed light on the hierarchy of factors impacting the selection of a pediatric dentist. It was found that the quality of the dental unit's disinfection process, the dentist's ability to manage uncooperative behaviour and communicate with the child, and the dentist's experience were paramount. Conversely, factors such as the dentist's gender, the proximity of the dental office to the child's school, and the number of pediatric dentists in the practice were less influential. These findings provide useful insights for dental professionals to understand parental priorities better and tailor their services accordingly. By focusing on the identified key factors, dental practices can enhance their appeal to parents, ultimately improving the oral health outcomes for children within the Kingdom of Saudi Arabia. Future research should consider utilizing a longitudinal design and exploring the interrelationship between various factors to provide an even more in-depth understanding of this complex decision-making process.

## Figures and Tables

**Table 1:** Detailed gender and age distribution of participating patients

**Gender**	**Age group (years)**	**Number of participants**	**Percentage (%)**
Male	20-30	51	9.4
	31-40	84	16
	41-50	86	16.2
Female	20-30	100	18.8
	31-40	110	20.7
	41-50	101	19

**Table 2:** Participants' residency and previous dental visits

**Region of residency**	**Previous dental visit?**	**Number of participants**	**Percentage (%)**
Riyadh	Yes	120	22.6
Riyadh	No	50	9.4
Jeddah	Yes	100	18.8
Jeddah	No	40	7.5
Other Regions	Yes	152	28.6
Other Regions	No	70	13.2

**Table 3:** Number of children per participant and concerns about dentists' treatment

**Number of children**	**Concern level (1-5 scale)**	**Number of participants**	**Percentage (%)**	**Mean concern level**	**SD**	**p-value**
1	1 (No concern)	20	3.8	1	0	-
1	3 (Moderate concern)	30	5.6	3	0	-
1	5 (High concern)	40	7.5	5	0	-
02-Mar	1 (No concern)	32	6	1	0	-
02-Mar	3 (Moderate concern)	88	16.5	3	0	-
02-Mar	5 (High concern)	60	11.3	5	0	-
4+	1 (No concern)	0	0	-	-	-
4+	3 (Moderate concern)	50	9.4	3	0	-
4+	5 (High concern)	0	0	-	-	-

**Table 4:** Tabular representation of the factors influencing the parents decision when selecting a paediatric dentist

**Factors**	**Very important**	**Somewhat important**	**Not very important**	**Not at all important**
Proximity to house	45.10%	32.70%	16.40%	5.80%
Opening hours	8.30%	30.80%	7.50%	3.40%
Short waiting time	71.20%	22.70%	4.50%	1.50%
Parking availability	58.50%	24.20%	13.20%	4.10%
Proximity to child's school	23.10%	15%	34.60%	27.30%
Amount of dental fee	69%	25%	3.60%	2.40%
Availability of up-to-date equipment and tools	81.40%	16.50%	1.70%	0.40%
Decoration at the dental office	35.90%	32.30%	22.90%	8.90%
Qualifications of pediatric dentist	59.80%	28%	9.60%	2.60%
Proof of continuous education of dentist	56.20%	25.90%	12.20%	5.60%
Dentist's experience	81.80%	15.80%	1.30%	1.10%
Number of pediatric dentists in practice	33.50%	29.50%	27.30%	9.80%
Child-friendly rooms and atmosphere	67.90%	23.50%	6.40%	2.30%
Play opportunities for children	41%	31.60%	18.80%	8.60%
Gender of pediatric dentist	22.70%	19.20%	26.50%	31.60%
Dentist's ability to communicate with child	83.80%	14.30%	1.10%	0.80%
Management of uncooperative behaviour in children	83.80%	14.10%	1.30%	0.80%
Quality of dental unit disinfection process	89.70%	8.30%	0.90%	1.10%

## References

[1] Adam TR (2022). Advances in preventive medicine..

[2] Kim MJ (2012). Journal of dental education.

[3] Mourad MS (2020). European Journal of Paediatric Dentistry..

[4] Volpato LER (2021). Brazilian Research in Pediatric Dentistry and Integrated Clinic..

[5] Lamprecht R (2020). Journal of oral rehabilitation..

[6] Agrawal A (2020). International Journal of Oral and Dental Health..

[7] Case A (2002). Health Aff (Millwood)..

[8] Murthy GA (2010). J Indian SocPedodPrev Dent..

[9] Grewal N (2007). J Indian SocPedodPrev Dent..

[10] Nepaul P (2020). J Int Soc Prev Community Dent..

[11] Kopczynski K (2021). Patient Prefer Adherence..

[12] Al-Mobeeriek A (2012). International journal of occupational medicine and environmental health..

[13] Kochar SP (2023). Cureus..

